# When monoclonal gammopathy‐associated chronic neutrophilic leukemia is a reactive process distinct from a clonal myeloproliferative neoplasm: Lessons from mistakes

**DOI:** 10.1002/jha2.713

**Published:** 2023-05-19

**Authors:** Christophe Willekens, Claude Chahine, Matteo Dragani, Sabine Khalife‐Hachem, Camille Bigenwald, Julien Rossignol, Cristina Castilla‐Llorente, Alina Danu, Jean‐Marie Michot, Veronique Saada, Sophie Cotteret, Christophe Marzac, Aline Renneville, Isabelle Plo, Sophie Broutin, Nelly Bosselut, Bruno Cassinat, Julien Lazarovici, Nathalie Droin, Stephane De Botton

**Affiliations:** ^1^ Département d'Hématologie Gustave Roussy Université Paris‐Saclay Villejuif France; ^2^ Inserm U1287 Gustave Roussy Université Paris‐Saclay Villejuif France; ^3^ Département d'Innovation Thérapeutique et d'Essais Précoces Gustave Roussy Université Paris‐Saclay Villejuif France; ^4^ Département de Biologie et Pathologie Médicales Gustave Roussy Université Paris‐Saclay Villejuif France; ^5^ AP‐HP Hôpital Saint‐Louis, Service de Biologie Cellulaire Paris France

**Keywords:** chronic neutrophilic leukemia, leukemoid reaction, leukocytosis, MGUS, proteasome inhibitor

1

Chronic neutrophilic leukemia (CNL) is a BCR::ABL1‐negative myeloproliferative neoplasm (MPN) characterized by sustained peripheral blood (PB) neutrophilia (white blood cell count ≥ 25×10^9^/L, with ≥80% neutrophils), bone marrow (BM) hypercellularity due to neutrophilic granulocyte proliferation without excess of blast or dysplasia. Somatic mutations of *CSF3R* gene, encoding the granulocyte colony stimulating factor (G‐CSF) receptor protein, are detected in >60% of cases and have significantly helped to confirm CNL diagnosis. Two classes of CSF3R mutations have been described (cytoplasmic domain truncations or membrane proximal threonine mutation as T618I or T640N), but all result in hyperactivation of the down‐stream Janus Kinase (JAK) signaling.[1, 2] Furthermore, *CSF3R* mutation could sensitize cell to tyrosine kinase inhibitors, such as ruxolitinib or dasatinib.

In the absence of a myeloid clonality such as *CSF3R* mutation, CNL diagnosis remains challenging. According to World Health Organization (WHO) classification, presence of a plasma cell disorders (PCDs) should be investigate to avoid CNL misdiagnosis.[3] In fact, numerous case studies have reported co‐existence of CNL and multiple myeloma/monoclonal gammopathy of unknown significance (MGUS).[4] In such cases, neutrophilia could be related to G‐CSF secretion from abnormal plasma cells (PCs), but confusion remains regarding shared clonality between MPN and PCD.[5, 6, 7]

Here, we report the case of an asymptomatic 56‐year‐old sub‐Saharian African woman, without relevant medical history, who was referred to explore a serum monoclonal IgA Lambda component (7.7 g/L) associated with hyperleucocytosis at 27.9 G/L with neutrophilia (81%) and monocytosis (9%). BM aspirate showed granular hyperplasia (84%) without significant dysplasia nor blast excess and a small percentage of dystrophic PC (1%). BM karyotype revealed a germline t(12;18)(q?24.3;q12) in all metaphases without *BCR::ABL* rearrangement by fluorescence in situ hybridization (FISH). BM biopsy revealed granulocytic hyperplasia without fibrosis. Whole body tomodensitometry did not detect any abnormality. A diagnosis of IgA Lambda MGUS was retained, without explanation about neutrophilia. Three years later, patient developed fatigue, bone pain, splenomegaly, and increased hyperleucocytosis (58.5G/L; neutrophils 88%). Serum monoclonal IgA Lambda component was stable at 7.3 g/L, and BM aspirate was unchanged. FISH did not reveal any *PDGFRA*, *PDGFRB*, or *FGFR1* rearrangement. Sequencing of 32 genes recurrently mutated in myeloid malignancies only revealed two rare single‐nucleotide polymorphisms (SNP) on *CSF3R* (p.R583H and p.T640I), predicted to be nondeleterious. Genetic analysis of nonhematopoietic tissues (nail and hair) confirmed germline origin of SNP p.T640I. Clonogenic assay was performed using purified PB CD34+ progenitors, as previously described.[8] In the absence of G‐CSF, a spontaneous growth of granulocyte colony‐forming unit (CFU‐G) progenitors was observed, and CFU‐G growth was partially reduced by JAK inhibition using ruxolitinib. Based on these results, a diagnosis of CNL without pathogenic *CSF3R* variant was retained at this time. Treatment with ruxolitinib and dasatinib were sequentially administered without efficacy. Due to increased leukocytosis associated with persistent systemic symptoms, patient received an allogeneic hematopoietic stem cell transplantation (AHSCT) from his human leukocyte antigen (HLA)‐identical healthy brother after sequential conditioning (clofarabine‐cytarabine followed by busulfan‐fludarabine‐ATG [anti‐thymocyte globulin]). At day 20 from AHSCT, hyperleucocytosis (85.6 G/L) mainly composed of neutrophils reoccurred, without receiving G‐CSF stimulation. BM aspirate showed persistent granular hyperplasia, but BM chimerism analysis surprisingly revealed that cells originated from donor (95% donor XY), arguing for an extrinsic stimulation, and that donor cells did not present the CSF3R T640I SNP (conversely to CSF3R R583H SNP). ELISA showed huge levels of G‐CSF in serum at 18300 pg/mL (N < 40). Serum monoclonal IgA Lambda was still detected (8 g/L) associated with 3% of BM PC. Overexpression of *CSF3* mRNA in purified BM CD138+ PC was confirmed by Quantitative Reverse transcription polymerase chain reaction (RTq‐PCR), in favor of G‐CSF production by recipient PC (Figure 1).

**FIGURE 1:**
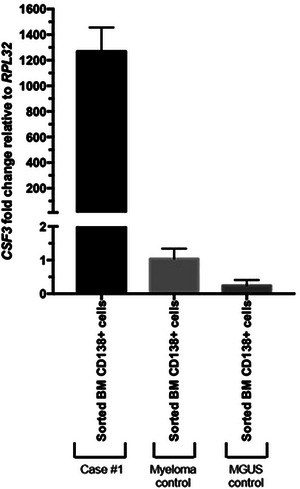
CSF3 mRNA expression by RTq‐PCR on purified bone marrow CD138+ plasma cells. CSF3, colony stimulating factor 3.

Based on these results, diagnosis was finally revised to reactive neutrophilia to PCD (MGUS), and patient subsequently received bortezomib‐dexamethasone (BD). After three cycles of 21‐days regimen, a drastic reduction of neutrophilia to 9.9 G/L was observed associated with a 32% spleen size reduction and decreased serum monoclonal component (2.7 g/L; 66% reduction). Concomitantly, a 37% serum G‐CSF level reduction was observed after two BD cycles (Figure 2). Unfortunately, treatment was discontinued due to adverse event. Five months later, leukocytosis reappeared (58 G/L), and patient received daratumumab‐dexamethasone without efficacy (Figure S1). Afterward, patient started a 3rd line of therapy with carfilzomib‐cyclophosphamide‐dexamethasone. After the first cycle, neutrophil count normalized associated with a severe thrombocytopenia (Figure S2). BM aspirate revealed a graft‐donor myelodysplastic syndrome (MDS) with excess of blast (12%), male karyotype with monosomy 7 associated with TET2 Q1545* (VAF 46%) and ETV6 R418K (VAF 45%) mutations. Of note, these mutations were not detected in blood and graft donor samples prior to transplantation. Patient concomitantly received carfilzomib‐dexamethasone and azacitidine. After 18 cycles of azacitidine, patient progressed to acute myeloid leukemia and died 4 months later.

**FIGURE 2:**
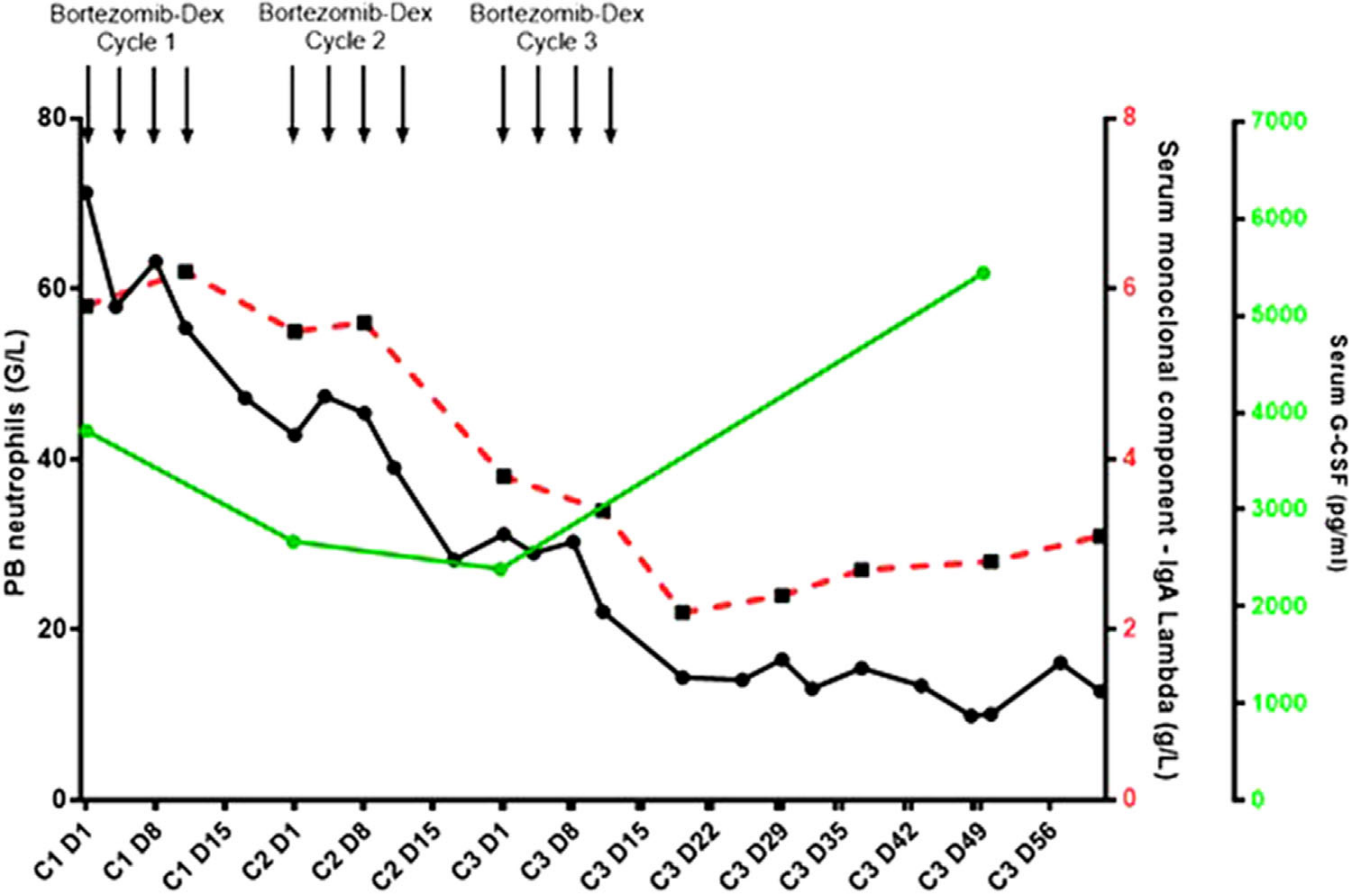
Peripheral blood (PB) neutrophils, serum monoclonal IgA Lambda component, and serum granulocyte colony stimulating factor (G‐CSF) evolution on bortezomib—dex. Black line, red dotted line, and green line represent PB neutrophils, serum IgA Lambda, and serum G‐CSF respectively.

Overall, donor leukocytosis after AHSCT with persistent monoclonal IgA lambda originating from recipient PC, *CSF3* mRNA overexpression in PC, high G‐CSF level on serum and response to PC‐targeted therapy strongly argue for a reactive neutrophilia induced by abnormal PC. This case confirms that clinicians should rule out CNL diagnosis in the absence of myeloid clonality when a PCD is detected.[3] In our patient, the presence of *CSF3R* SNP in a pathogenic position (T640, previously named T617) was misleading, but BM donor cells reproduced the CNL phenotype while being negative for CSF3R T640I SNP, which strongly supports his nonpathogenic role.[8, 9]. Results of clonogenic assays were also misleading and probably related to the extremely high G‐CSF levels. In fact, serum G‐CSF dosage could be the most powerful tool to make a prompt diagnosis of reactive neutrophilia to PCD which could have avoid, in our patient, inefficient therapy (as ruxolitinib), AHSCT morbidity, and donor‐cell MDS.

The hypothesis that CNL and PCD diagnosis could be concomitant inspired a “monoclonal gammopathy‐associated CNL” entity.[6] It is currently not clear whether the CNL and PCD are clonally related, but phenocopy observed after AHSCT suggests a nonclonal relationship in our case. Our patient also presented a donor‐cell MDS, a rare condition after AHSCT.[10] Thus, we could hypothesize that continuous exposition to high level of G‐CSF may promote development of a bona fide clonal myeloid disease, which could explain concomitant diagnosis in previous reports.

Finally, this report describes a correlated response to BD between PB neutrophils, spleen size, monoclonal component, and serum G‐CSF level.[11, 12] These results suggest that PC‐targeted therapy is efficient in such case and may limit the risk of clonal evolution into a myeloid malignancy by decreasing G‐CSF production.

## AUTHOR CONTRIBUTIONS

CW, CC, MD, SKH, CB, JR, CCL, JMM, AD, JL, and SDB followed the patient. VS performed cytological analysis. SC performed cytogenetic analysis. CM and AR supervised molecular analysis. IP supervised CFU‐G assay. ND and CW performed RTqPCR assay. SB, NB, and BC supervised and performed G‐CSF dosage. CW collected data and interpreted the results. CW wrote the manuscript. CB, JR, CCL, JMM, VS, SC, AR, IP, SB, ND, and SDB revised the manuscript. All authors approved the manuscript.

## CONFLICT OF INTEREST STATEMENT

The authors have no conflict of interest.

## FUNDING INFORMATION

The authors received no specific funding for this work.

## ETHICS STATEMENT

Institutional review board authorized report. The patient provided informed consent to germline genetic analysis and publication of clinical and genetic data.

## Supporting information

Supporting Information

Supporting Information
