# Supplementary material for: When monoclonal gammopathy‐associated chronic neutrophilic leukemia is a reactive process distinct from a clonal myeloproliferative neoplasm: Lessons from mistakes
Source: EJHaem. 2023 May 19;4(3):823–6. doi: 10.1002/jha2.713 (PMC10435719; doi:10.1002/jha2.713)
Supplement: Supplementary file 1 — Supporting Information [file JHA2-4-823-s002.docx]

**Supplemental informations**

- 1. ***Molecular analysis of gene mutation by NGS.***

Genomic DNA was assessed for mutations in 34 genes (*ASXL1, BCOR, BCORL1, CALR, CBL, CEBPA, CSF3R, DNMT3A, ETV6, EZH2, FLT3-*TKD*, IDH1, IDH2, JAK2, KIT, KRAS, MPL, NPM1, NRAS, PHF6, PTPN11, RIT1, RUNX1, SF3B1, SRSF2, STAG2, TET2, TP53, U2AF1, WT1* and *ZRSR2*) by Next-generation sequencing (NGS) using the Ion AmpliSeq Library Kit 2.0 (384 reactions; Thermo FisherScientific, Carlsbad, CA). Multiplex PCR amplifications were performed from 2 x 5 ng of genomic DNA (pool 1: 284 primer pairs; pool 2: 274 primer pairs). After amplification, barcodes and adaptors were added to amplicons by ligation. Products were subjected to a selective purification on AMPure beads (BeckmanCoulter, Brea,CA). Emulsion polymerase chain reaction (PCR) was performed using the Ion Chef instrument and the Ion 540 Kit-Chef (Thermo Fisher Scientific). Sequencing was performed with the S5XL system (Thermo Fisher Scientific) onto the Ion 540 chip (40 samples per chip). For bioinformatic analysis, base calls were generated by the Torrent Browser software (5.0.4 version) using the included variant caller with an additional plug-in (Thermo Fisher Scientific). The .bam and.vcf files were used for further analysis. The .vcf files were annotated with the Ion reporter software (Thermo Fisher Scientific) and processed for a second analysis of the indexed files using the Sequence Pilot software (4.2.1 version) (JSI Medical Systems, Ettenheim, Germany). Results were compared with selection of variants that will be further considered. For each variant, depth at the variant position (number of mutated reads and unmutated reads) was considered to calculate variant allele frequency (VAF), which is the proportion of mutated reads among total reads. Minimum reportable VAF is 2% of sequencing depth at least 100X.

| *Gene* | Reference sequence | Exons analyzed anaanalyzscreened |
| --- | --- | --- |
| *ASXL1* | NM_015338 | 11 and 12 |
| *BCOR* | NM_001123385 | 2 to 15 |
| *BCORL1* | NM_021946 | 1 to 12 |
| *CALR* | NM_004343 | 9 |
| *CBL* | NM_005188 | 8 and 9 |
| ***CSF3R*** | NM_156039 | **14 to 17** |
| *DNMT3A* | NM_022552 | 2 to 23 |
| *ETV6* | NM_001987 | 1 to 8 |
| *EZH2* | NM_004456 | 2 to 20 |
| *FLT3-TKD* | NM_004119 | 20 |
| *GATA2* | NM_032638 | 2 to 6 |
| *IDH1* | NM_005896 | 4 |
| *IDH2* | NM_002168 | 4 |
| *JAK2* | NM_004972 | 12 and 14 |
| *KIT* | NM_000222 | 8 to 11, 17 |
| *KRAS* | NM_033360 | 2 and 3 |
| *MPL* | NM_005373 | 10 |
| *NPM1* | NM_002520 | 11 |
| *NRAS* | NM_002524 | 2 and 3 |
| *PHF6* | NM_001015877 | 2 to 10 |
| *PTPN11* | NM_002834 | 3 and 13 |
| *RIT1* | NM_006912 | 5 |
| *RUNX1* | NM_001001890 | 1 to 6 |
| *SETBP1* | NM_015559 | 4 |
| *SF3B1* | NM_012433 | 13 to 16 |
| *SRSF2* | NM_003016 | 1 |
| *STAG2* | NM_001042749 | 3 to 35 |
| *TET2* | NM_001127208 | 3 to 11 |
| *TP53* | NM_001126112 | 3 to 11 |
| *U2AF1* | NM_006758 | 2 and 6 |
| *WT1* | NM_024426 | 7 and 9 |
| *ZRSR2* | NM_005089 | 1 to 11 |

- 1. **RTq-PCR on purified CD138+ bone marrow cells:**

Total RNA was obtained from frozen dry pellet of sorted cells using Trizol™ Reagent (Invitrogen Thermo Fischer) and Direct-zol™ RNA Miniprep (Zymo research). Total RNA was reverse transcribed with SuperScript IV reverse transcriptase with random hexamers (Thermo Fisher Scientific). Real-time quantitative polymerase chain reaction (RT-qPCR) was performed with AmpliTaq Gold polymerase in SYBR Green master mix using an Applied Biosystems 7500 thermocycler (Thermo Fisher Scientific).Primers used are:

human RPL32 (F: 5'-TGTCCTGAATGTGGTCACCTGA-3'; R: 5'-CTGCAGTCTCCTTGCACACCT-3') and human CSF3 (F: 5'-GAGTTGGGTCCCACCTTG-3'; R: 5'-TGGAAAGCAGAGGCGAAG-3').
