# Supplementary material for: When monoclonal gammopathy‐associated chronic neutrophilic leukemia is a reactive process distinct from a clonal myeloproliferative neoplasm: Lessons from mistakes
Source: EJHaem. 2023 May 19;4(3):823–6. doi: 10.1002/jha2.713 (PMC10435719; doi:10.1002/jha2.713)

Supplemental Figure 1: Peripheral blood neutrophils and monoclonal IgA Lambda component evolution on daratumumab – dexamethasone. Black line and grey line represent PB neutrophils and serum IgA Lambda.

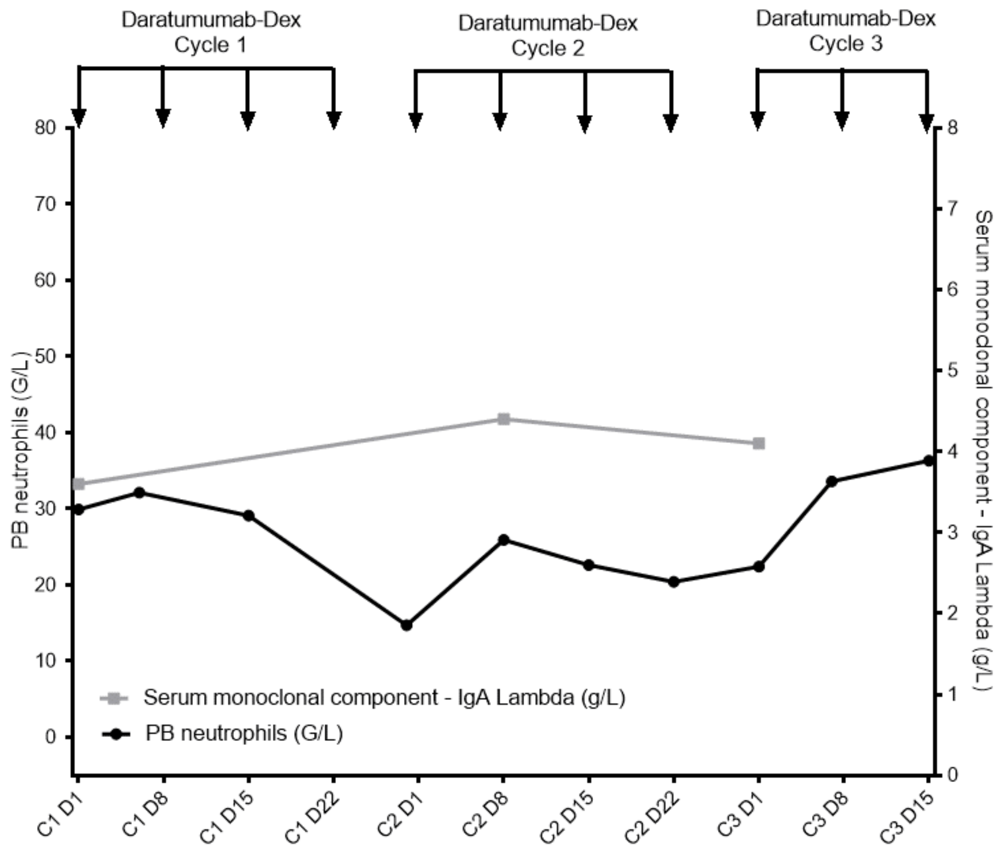

Supplemental Figure 2: Peripheral blood neutrophils and monoclonal IgA Lambda component evolution on carfilzomib – dexamethasone. Black line and grey line represent PB neutrophils and serum IgA Lambda.

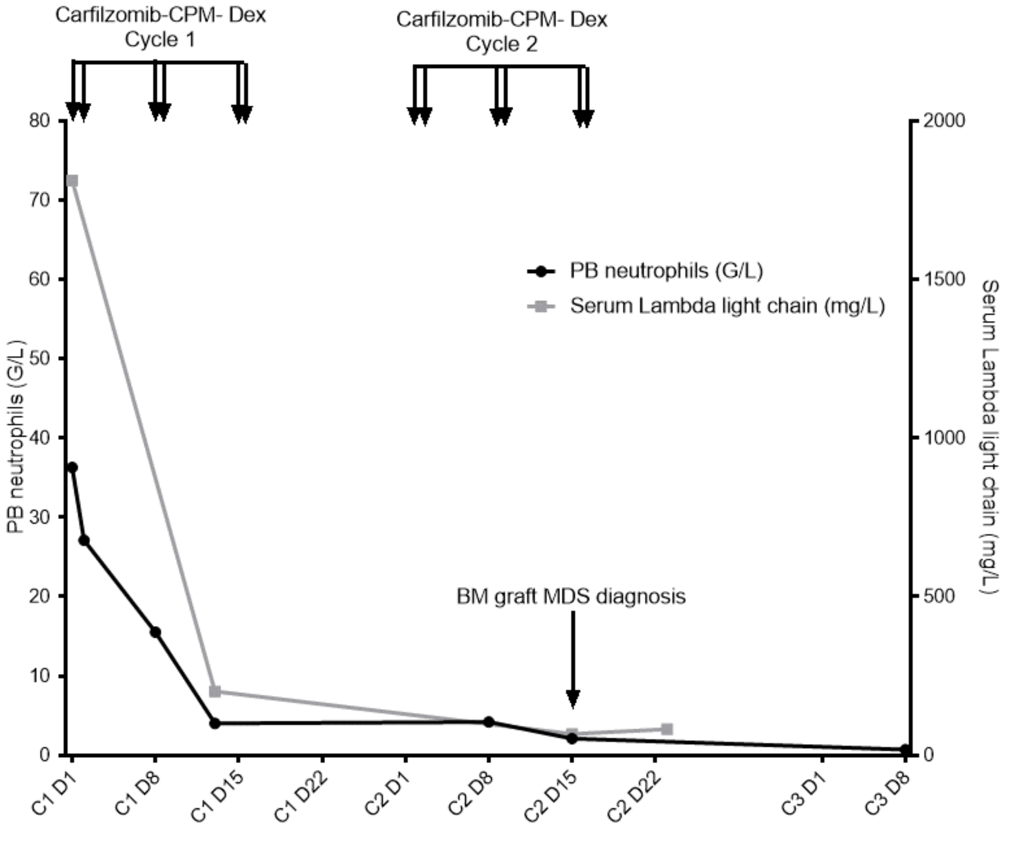

Supplement: Supplementary file 2 — Supporting Information [file JHA2-4-823-s001.pdf]
